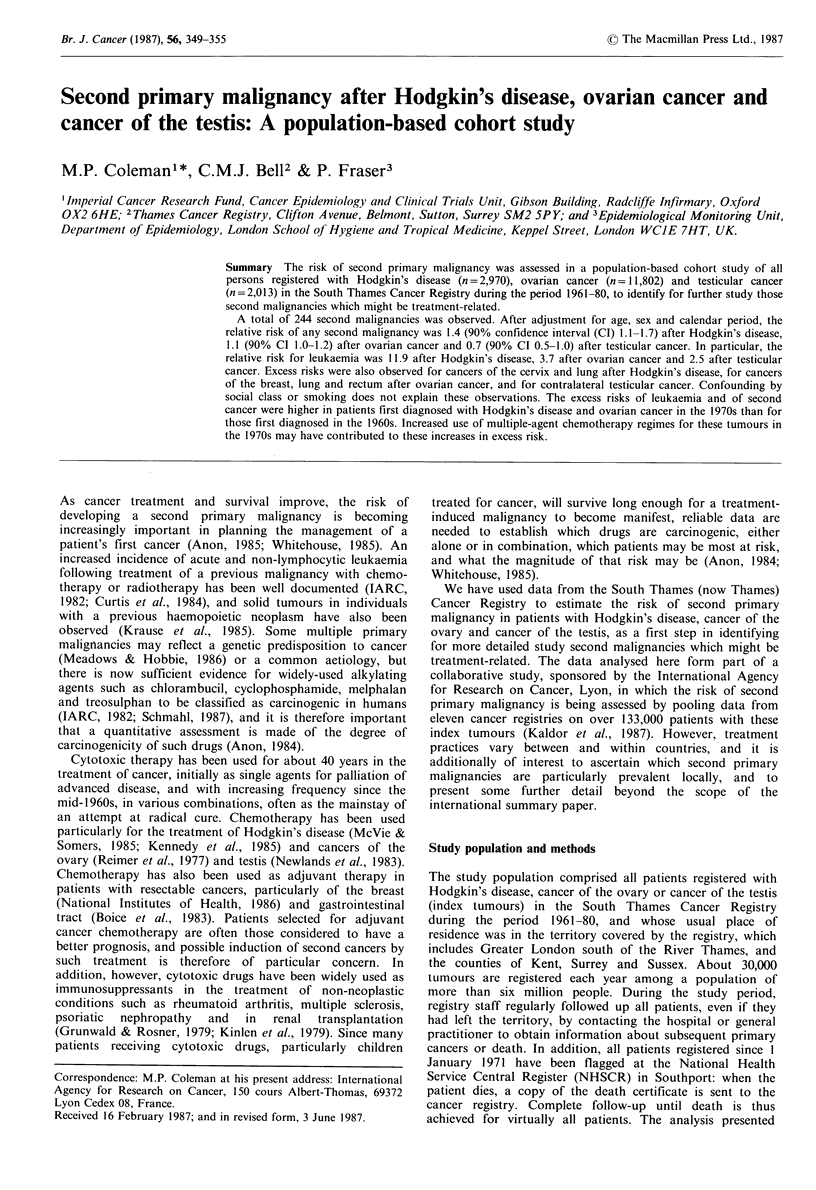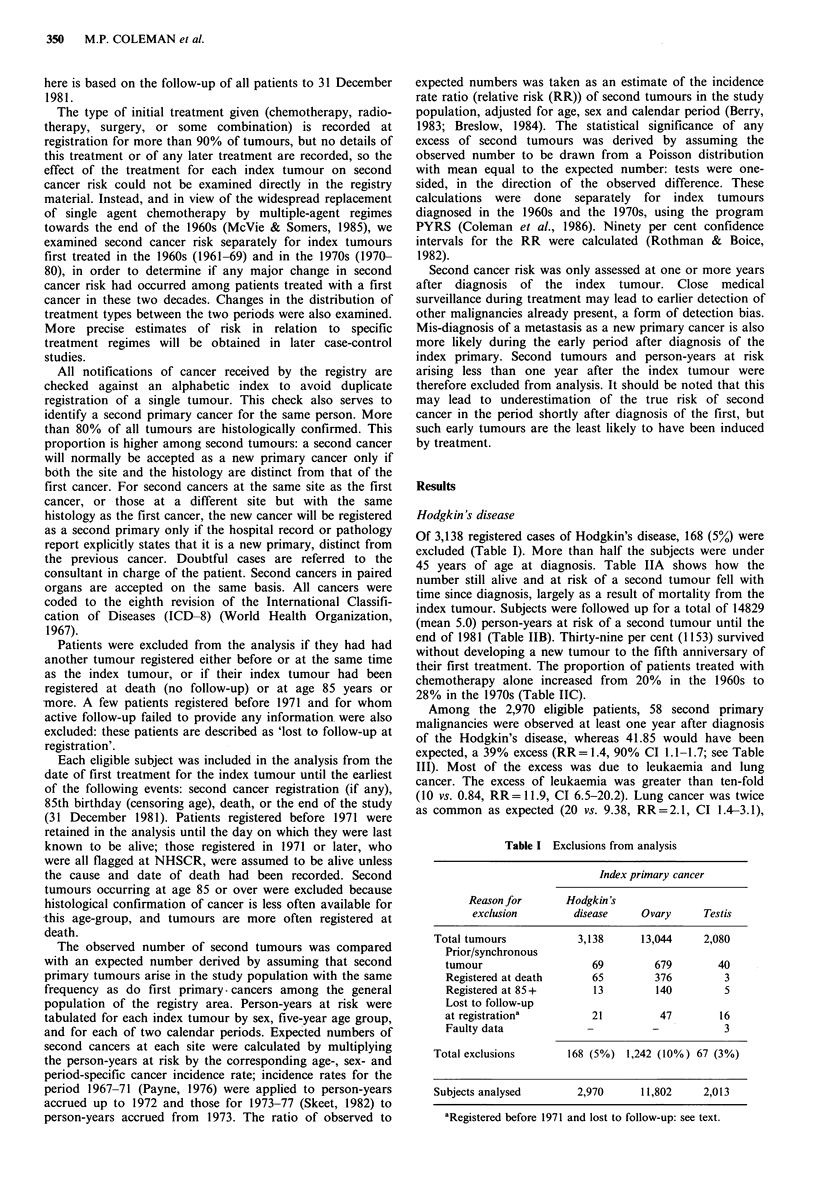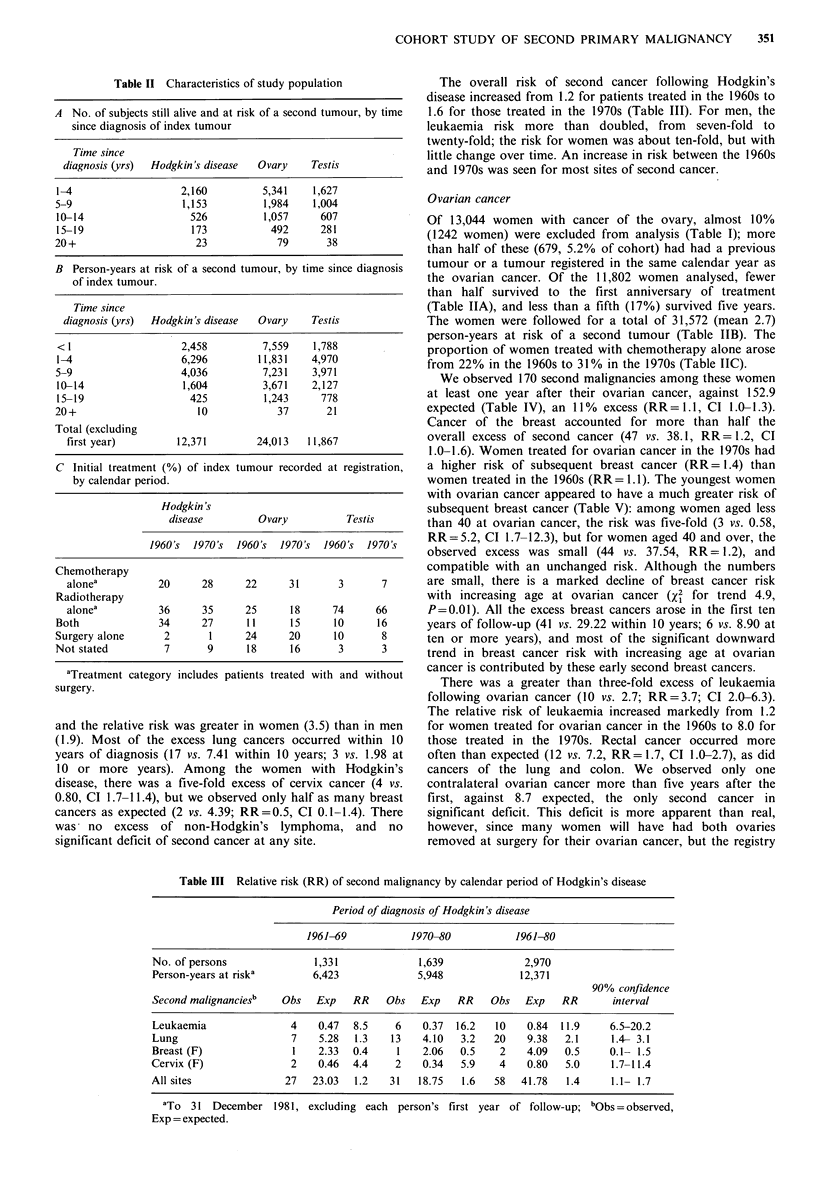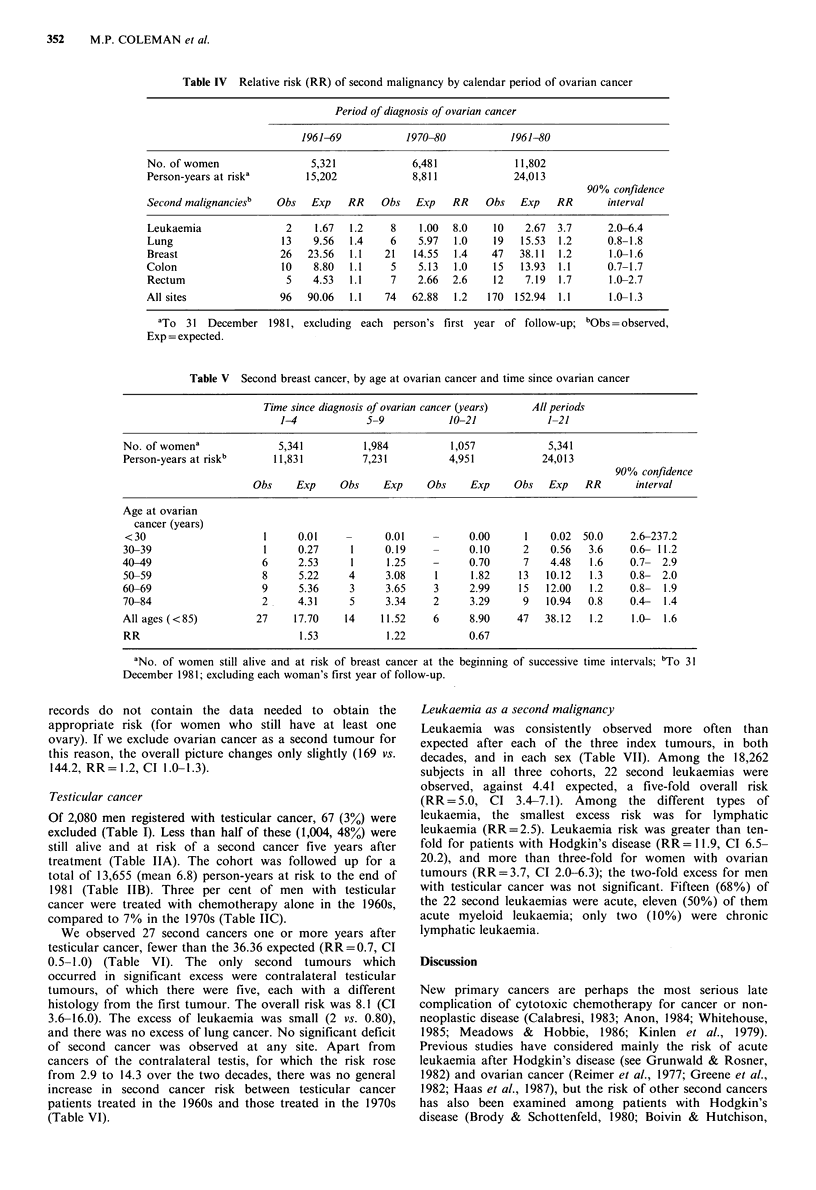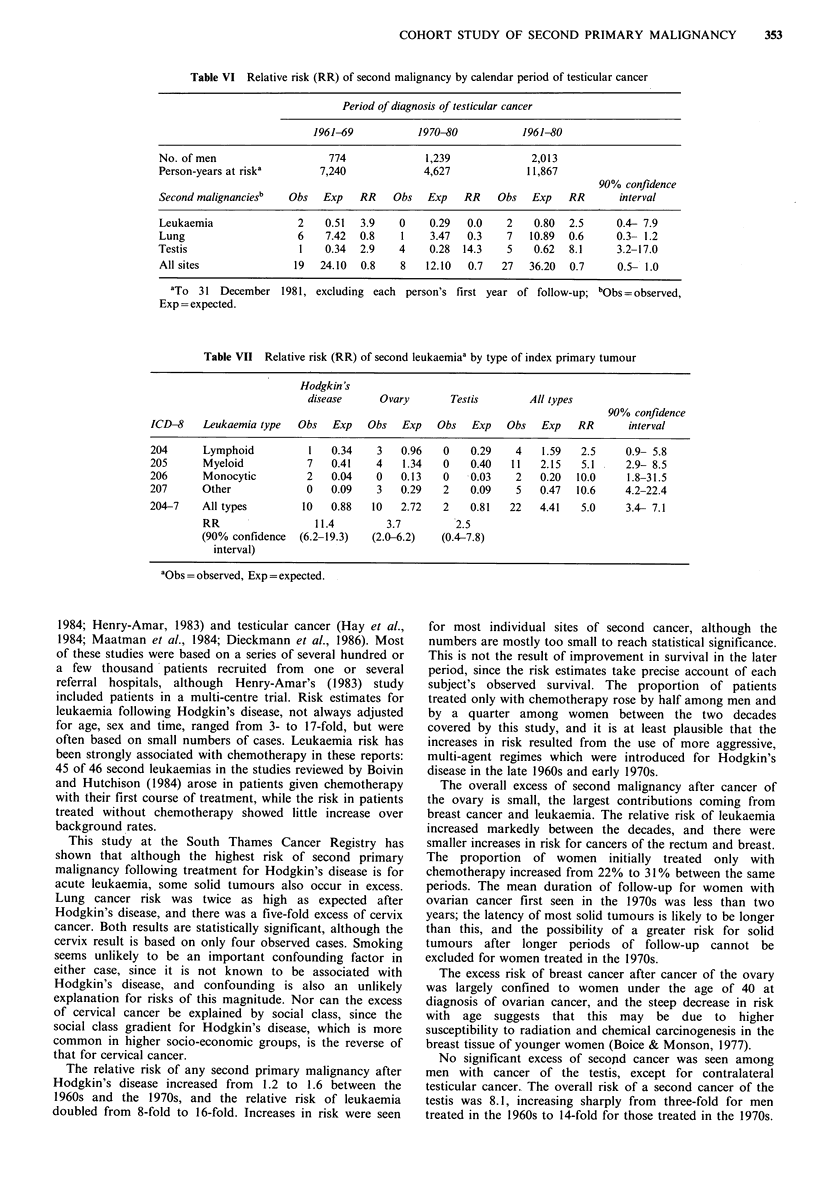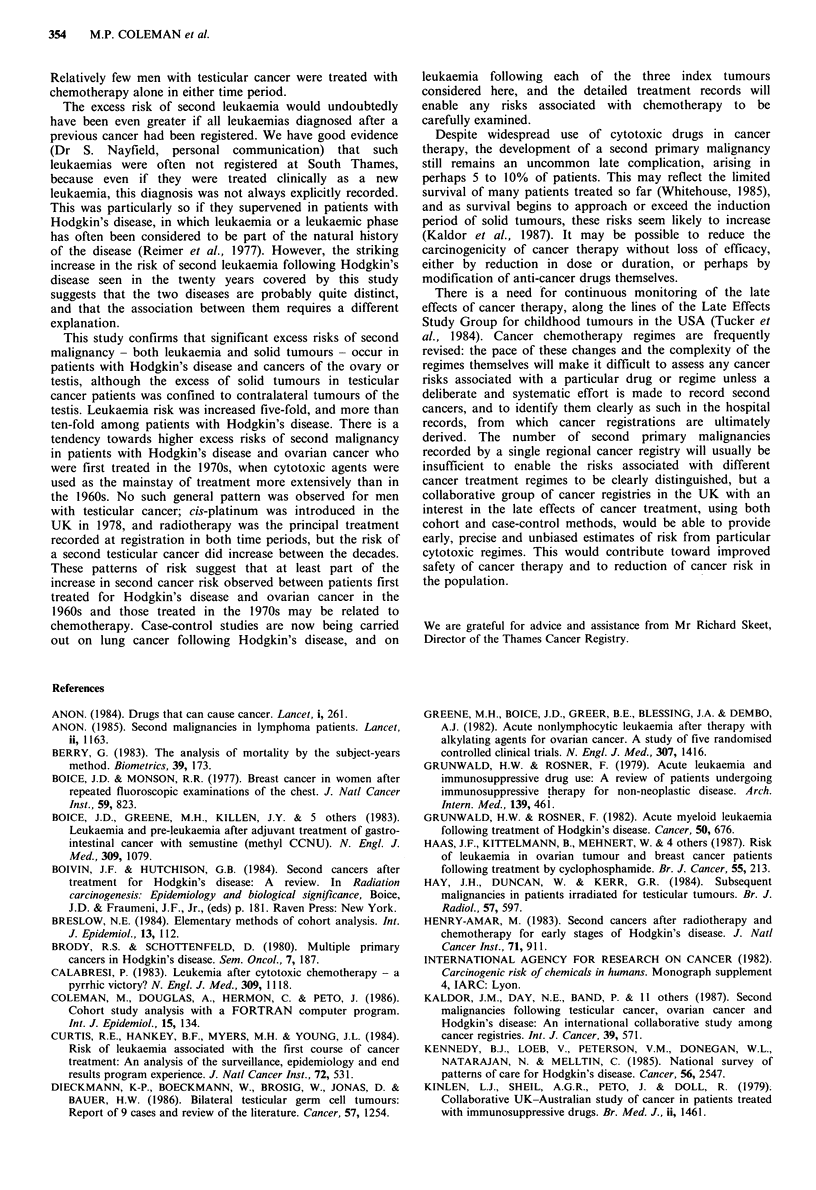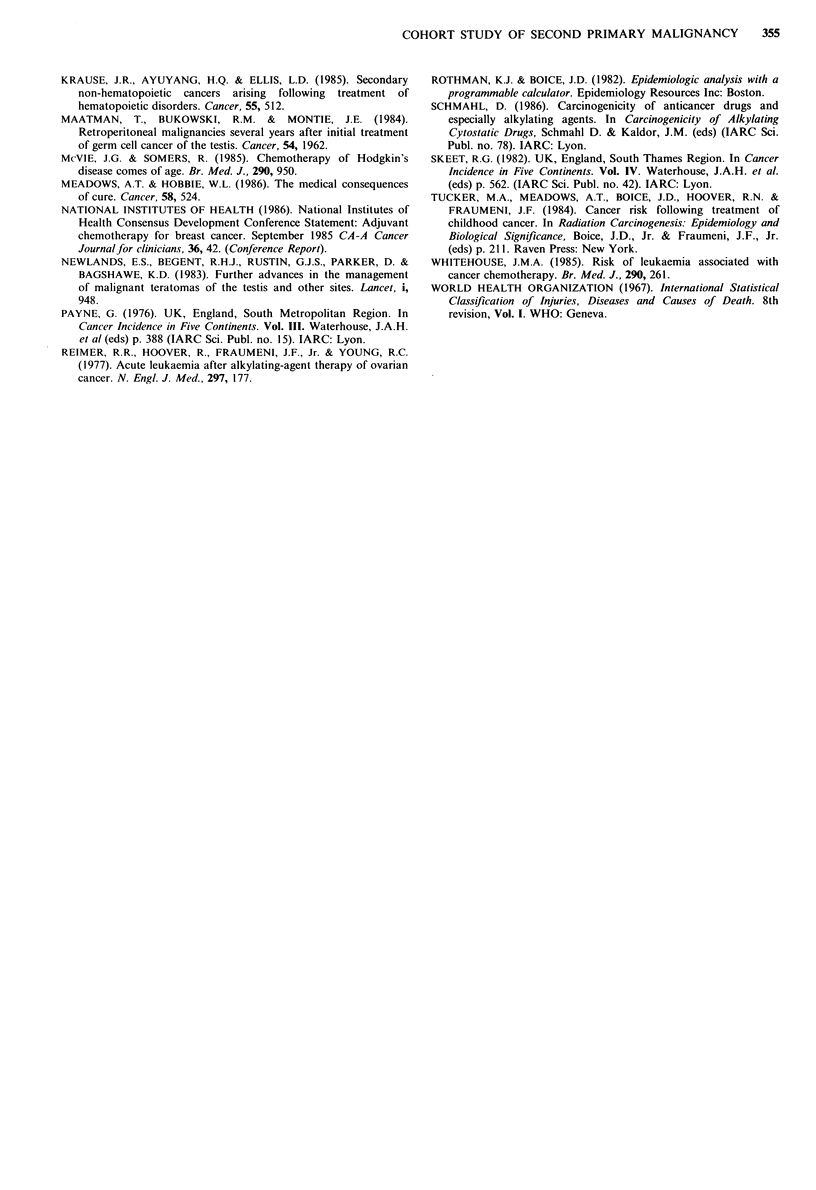# Second primary malignancy after Hodgkin's disease, ovarian cancer and cancer of the testis: a population-based cohort study.

**DOI:** 10.1038/bjc.1987.201

**Published:** 1987-09

**Authors:** M. P. Coleman, C. M. Bell, P. Fraser

**Affiliations:** Imperial Cancer Research Fund, Cancer Epidemiology and Clinical Trials Unit, Radcliffe Infirmary, Oxford.

## Abstract

The risk of second primary malignancy was assessed in a population-based cohort study of all persons registered with Hodgkin's disease (n = 2,970), ovarian cancer (n = 11,802) and testicular cancer (n = 2,013) in the South Thames Cancer Registry during the period 1961-80, to identify for further study those second malignancies which might be treatment-related. A total of 244 second malignancies was observed. After adjustment for age, sex and calendar period, the relative risk of any second malignancy was 1.4 (90% confidence interval (CI) 1.1-1.7) after Hodgkin's disease, 1.1 (90% CI 1.0-1.2) after ovarian cancer and 0.7 (90% CI 0.5-1.0) after testicular cancer. In particular, the relative risk for leukaemia was 11.9 after Hodgkin's disease, 3.7 after ovarian cancer and 2.5 after testicular cancer. Excess risks were also observed for cancers of the cervix and lung after Hodgkin's disease, for cancers of the breast, lung and rectum after ovarian cancer, and for contralateral testicular cancer. Confounding by social class or smoking does not explain these observations. The excess risks of leukaemia and of second cancer were higher in patients first diagnosed with Hodgkin's disease and ovarian cancer in the 1970s than for those first diagnosed in the 1960s. Increased use of multiple-agent chemotherapy regimes for these tumours in the 1970s may have contributed to these increases in excess risk.


Br. J. Cancer (1987), 56, 349–355 © The Macmillan Press Ltd., 1987

### Second primary malignancy after Hodgkin's disease, ovarian cancer and cancer of the testis: A population-based cohort study

M.P. Coleman[1]*, C.M.J. Bell[2] & P. Fraser[3]

[1]*Imperial Cancer Research Fund, Cancer Epidemiology and Clinical Trials Unit, Gibson Building, Radcliffe Infirmary, Oxford OX2 6HE;* [2]*Thames Cancer Registry, Clifton Avenue, Belmont, Sutton, Surrey SM2 5PY; and* [3]*Epidemiological Monitoring Unit, Department of Epidemiology, London School of Hygiene and Tropical Medicine, Keppel Street, London WC1E 7HT, UK.*

**Summary** The risk of second primary malignancy was assessed in a population-based cohort study of all persons registered with Hodgkin's disease ($n$=2,970), ovarian cancer ($n$=11,802) and testicular cancer ($n$=2,013) in the South Thames Cancer Registry during the period 1961–80, to identify for further study those second malignancies which might be treatment-related.

A total of 244 second malignancies was observed. After adjustment for age, sex and calendar period, the relative risk of any second malignancy was 1.4 (90% confidence interval (CI) 1.1–1.7) after Hodgkin's disease, 1.1 (90% CI 1.0–1.2) after ovarian cancer and 0.7 (90% CI 0.5–1.0) after testicular cancer. In particular, the relative risk for leukaemia was 11.9 after Hodgkin's disease, 3.7 after ovarian cancer and 2.5 after testicular cancer. Excess risks were also observed for cancers of the cervix and lung after Hodgkin's disease, for cancers of the breast, lung and rectum after ovarian cancer, and for contralateral testicular cancer. Confounding by social class or smoking does not explain these observations. The excess risks of leukaemia and of second cancer were higher in patients first diagnosed with Hodgkin's disease and ovarian cancer in the 1970s than for those first diagnosed in the 1960s. Increased use of multiple-agent chemotherapy regimes for these tumours in the 1970s may have contributed to these increases in excess risk.

As cancer treatment and survival improve, the risk of developing a second primary malignancy is becoming increasingly important in planning the management of a patient's first cancer (Anon, 1985; Whitehouse, 1985). An increased incidence of acute and non-lymphocytic leukaemia following treatment of a previous malignancy with chemotherapy or radiotherapy has been well documented (IARC, 1982; Curtis *et al.*, 1984), and solid tumours in individuals with a previous haemopoietic neoplasm have also been observed (Krause *et al.*, 1985). Some multiple primary malignancies may reflect a genetic predisposition to cancer (Meadows & Hobbie, 1986) or a common aetiology, but there is now sufficient evidence for widely-used alkylating agents such as chlorambucil, cyclophosphamide, melphalan and treosulphan to be classified as carcinogenic in humans (IARC, 1982; Schmahl, 1987), and it is therefore important that a quantitative assessment is made of the degree of carcinogenicity of such drugs (Anon, 1984).

Cytotoxic therapy has been used for about 40 years in the treatment of cancer, initially as single agents for palliation of advanced disease, and with increasing frequency since the mid-1960s, in various combinations, often as the mainstay of an attempt at radical cure. Chemotherapy has been used particularly for the treatment of Hodgkin's disease (McVie & Somers, 1985; Kennedy *et al.*, 1985) and cancers of the ovary (Reimer *et al.*, 1977) and testis (Newlands *et al.*, 1983). Chemotherapy has also been used as adjuvant therapy in patients with resectable cancers, particularly of the breast (National Institutes of Health, 1986) and gastrointestinal tract (Boice *et al.*, 1983). Patients selected for adjuvant cancer chemotherapy are often those considered to have a better prognosis, and possible induction of second cancers by such treatment is therefore of particular concern. In addition, however, cytotoxic drugs have been widely used as immunosuppressants in the treatment of non-neoplastic conditions such as rheumatoid arthritis, multiple sclerosis, psoriatic nephropathy and in renal transplantation (Grunwald & Rosner, 1979; Kinlen *et al.*, 1979). Since many patients receiving cytotoxic drugs, particularly children treated for cancer, will survive long enough for a treatment-induced malignancy to become manifest, reliable data are needed to establish which drugs are carcinogenic, either alone or in combination, which patients may be most at risk, and what the magnitude of that risk may be (Anon, 1984; Whitehouse, 1985).

We have used data from the South Thames (now Thames) Cancer Registry to estimate the risk of second primary malignancy in patients with Hodgkin's disease, cancer of the ovary and cancer of the testis, as a first step in identifying for more detailed study second malignancies which might be treatment-related. The data analysed here form part of a collaborative study, sponsored by the International Agency for Research on Cancer, Lyon, in which the risk of second primary malignancy is being assessed by pooling data from eleven cancer registries on over 133,000 patients with these index tumours (Kaldor *et al.*, 1987). However, treatment practices vary between and within countries, and it is additionally of interest to ascertain which second primary malignancies are particularly prevalent locally, and to present some further detail beyond the scope of the international summary paper.

### Study population and methods

The study population comprised all patients registered with Hodgkin's disease, cancer of the ovary or cancer of the testis (index tumours) in the South Thames Cancer Registry during the period 1961–80, and whose usual place of residence was in the territory covered by the registry, which includes Greater London south of the River Thames, and the counties of Kent, Surrey and Sussex. About 30,000 tumours are registered each year among a population of more than six million people. During the study period, registry staff regularly followed up all patients, even if they had left the territory, by contacting the hospital or general practitioner to obtain information about subsequent primary cancers or death. In addition, all patients registered since 1 January 1971 have been flagged at the National Health Service Central Register (NHSCR) in Southport: when the patient dies, a copy of the death certificate is sent to the cancer registry. Complete follow-up until death is thus achieved for virtually all patients. The analysis presented

Correspondence: M.P. Coleman at his present address: International Agency for Research on Cancer, 150 cours Albert-Thomas, 69372 Lyon Cedex 08, France.
Received 16 February 1987; and in revised form, 3 June 1987.

here is based on the follow-up of all patients to 31 December 1981.

The type of initial treatment given (chemotherapy, radiotherapy, surgery, or some combination) is recorded at registration for more than 90% of tumours, but no details of this treatment or of any later treatment are recorded, so the effect of the treatment for each index tumour on second cancer risk could not be examined directly in the registry material. Instead, and in view of the widespread replacement of single agent chemotherapy by multiple-agent regimes towards the end of the 1960s (McVie & Somers, 1985), we examined second cancer risk separately for index tumours first treated in the 1960s (1961–69) and in the 1970s (1970–80), in order to determine if any major change in second cancer risk had occurred among patients treated with a first cancer in these two decades. Changes in the distribution of treatment types between the two periods were also examined. More precise estimates of risk in relation to specific treatment regimes will be obtained in later case-control studies.

All notifications of cancer received by the registry are checked against an alphabetic index to avoid duplicate registration of a single tumour. This check also serves to identify a second primary cancer for the same person. More than 80% of all tumours are histologically confirmed. This proportion is higher among second tumours: a second cancer will normally be accepted as a new primary cancer only if both the site and the histology are distinct from that of the first cancer. For second cancers at the same site as the first cancer, or those at a different site but with the same histology as the first cancer, the new cancer will be registered as a second primary only if the hospital record or pathology report explicitly states that it is a new primary, distinct from the previous cancer. Doubtful cases are referred to the consultant in charge of the patient. Second cancers in paired organs are accepted on the same basis. All cancers were coded to the eighth revision of the International Classification of Diseases (ICD-8) (World Health Organization, 1967).

Patients were excluded from the analysis if they had had another tumour registered either before or at the same time as the index tumour, or if their index tumour had been registered at death (no follow-up) or at age 85 years or more. A few patients registered before 1971 and for whom active follow-up failed to provide any information were also excluded: these patients are described as 'lost to follow-up at registration'.

Each eligible subject was included in the analysis from the date of first treatment for the index tumour until the earliest of the following events: second cancer registration (if any), 85th birthday (censoring age), death, or the end of the study (31 December 1981). Patients registered before 1971 were retained in the analysis until the day on which they were last known to be alive; those registered in 1971 or later, who were all flagged at NHSCR, were assumed to be alive unless the cause and date of death had been recorded. Second tumours occurring at age 85 or over were excluded because histological confirmation of cancer is less often available for this age-group, and tumours are more often registered at death.

The observed number of second tumours was compared with an expected number derived by assuming that second primary tumours arise in the study population with the same frequency as do first primary cancers among the general population of the registry area. Person-years at risk were tabulated for each index tumour by sex, five-year age group, and for each of two calendar periods. Expected numbers of second cancers at each site were calculated by multiplying the person-years at risk by the corresponding age-, sex- and period-specific cancer incidence rate; incidence rates for the period 1967–71 (Payne, 1976) were applied to person-years accrued up to 1972 and those for 1973–77 (Skeet, 1982) to person-years accrued from 1973. The ratio of observed to expected numbers was taken as an estimate of the incidence rate ratio (relative risk (RR)) of second tumours in the study population, adjusted for age, sex and calendar period (Berry, 1983; Breslow, 1984). The statistical significance of any excess of second tumours was derived by assuming the observed number to be drawn from a Poisson distribution with mean equal to the expected number: tests were one-sided, in the direction of the observed difference. These calculations were done separately for index tumours diagnosed in the 1960s and the 1970s, using the program PYRS (Coleman *et al.*, 1986). Ninety per cent confidence intervals for the RR were calculated (Rothman & Boice, 1982).

Second cancer risk was only assessed at one or more years after diagnosis of the index tumour. Close medical surveillance during treatment may lead to earlier detection of other malignancies already present, a form of detection bias. Mis-diagnosis of a metastasis as a new primary cancer is also more likely during the early period after diagnosis of the index primary. Second tumours and person-years at risk arising less than one year after the index tumour were therefore excluded from analysis. It should be noted that this may lead to underestimation of the true risk of second cancer in the period shortly after diagnosis of the first, but such early tumours are the least likely to have been induced by treatment.

### Results

### *Hodgkin's disease*

Of 3,138 registered cases of Hodgkin's disease, 168 (5%) were excluded (Table I). More than half the subjects were under 45 years of age at diagnosis. Table IIA shows how the number still alive and at risk of a second tumour fell with time since diagnosis, largely as a result of mortality from the index tumour. Subjects were followed up for a total of 14829 (mean 5.0) person-years at risk of a second tumour until the end of 1981 (Table IIB). Thirty-nine per cent (1153) survived without developing a new tumour to the fifth anniversary of their first treatment. The proportion of patients treated with chemotherapy alone increased from 20% in the 1960s to 28% in the 1970s (Table IIC).

Among the 2,970 eligible patients, 58 second primary malignancies were observed at least one year after diagnosis of the Hodgkin's disease, whereas 41.85 would have been expected, a 39% excess (RR = 1.4, 90% CI 1.1–1.7; see Table III). Most of the excess was due to leukaemia and lung cancer. The excess of leukaemia was greater than ten-fold (10 *vs.* 0.84, RR = 11.9, CI 6.5–20.2). Lung cancer was twice as common as expected (20 *vs.* 9.38, RR = 2.1, CI 1.4–3.1),

**Table I** Exclusions from analysis

| *Reason for exclusion* | *Index primary cancer* | | |
|---|---|---|---|
| | *Hodgkin's disease* | *Ovary* | *Testis* |
| Total tumours | 3,138 | 13,044 | 2,080 |
| Prior/synchronous tumour | 69 | 679 | 40 |
| Registered at death | 65 | 376 | 3 |
| Registered at 85+ | 13 | 140 | 5 |
| Lost to follow-up at registration[a] | 21 | 47 | 16 |
| Faulty data | – | – | 3 |
| Total exclusions | 168 (5%) | 1,242 (10%) | 67 (3%) |
| Subjects analysed | 2,970 | 11,802 | 2,013 |

[a]Registered before 1971 and lost to follow-up: see text.

**Table II** Characteristics of study population

*A* No. of subjects still alive and at risk of a second tumour, by time since diagnosis of index tumour

| *Time since diagnosis (yrs)* | *Hodgkin's disease* | *Ovary* | *Testis* |
|---|---|---|---|
| 1–4 | 2,160 | 5,341 | 1,627 |
| 5–9 | 1,153 | 1,984 | 1,004 |
| 10–14 | 526 | 1,057 | 607 |
| 15–19 | 173 | 492 | 281 |
| 20+ | 23 | 79 | 38 |

*B* Person-years at risk of a second tumour, by time since diagnosis of index tumour.

| *Time since diagnosis (yrs)* | *Hodgkin's disease* | *Ovary* | *Testis* |
|---|---|---|---|
| <1 | 2,458 | 7,559 | 1,788 |
| 1–4 | 6,296 | 11,831 | 4,970 |
| 5–9 | 4,036 | 7,231 | 3,971 |
| 10–14 | 1,604 | 3,671 | 2,127 |
| 15–19 | 425 | 1,243 | 778 |
| 20+ | 10 | 37 | 21 |
| Total (excluding first year) | 12,371 | 24,013 | 11,867 |

*C* Initial treatment (%) of index tumour recorded at registration, by calendar period.

| | *Hodgkin's disease* | | *Ovary* | | *Testis* | |
|---|---|---|---|---|---|---|
| | *1960's* | *1970's* | *1960's* | *1970's* | *1960's* | *1970's* |
| Chemotherapy alone[a] | 20 | 28 | 22 | 31 | 3 | 7 |
| Radiotherapy alone[a] | 36 | 35 | 25 | 18 | 74 | 66 |
| Both | 34 | 27 | 11 | 15 | 10 | 16 |
| Surgery alone | 2 | 1 | 24 | 20 | 10 | 8 |
| Not stated | 7 | 9 | 18 | 16 | 3 | 3 |

[a]Treatment category includes patients treated with and without surgery.

and the relative risk was greater in women (3.5) than in men (1.9). Most of the excess lung cancers occurred within 10 years of diagnosis (17 *vs.* 7.41 within 10 years; 3 *vs.* 1.98 at 10 or more years). Among the women with Hodgkin's disease, there was a five-fold excess of cervix cancer (4 *vs.* 0.80, CI 1.7–11.4), but we observed only half as many breast cancers as expected (2 *vs.* 4.39; RR=0.5, CI 0.1–1.4). There was no excess of non-Hodgkin's lymphoma, and no significant deficit of second cancer at any site.

The overall risk of second cancer following Hodgkin's disease increased from 1.2 for patients treated in the 1960s to 1.6 for those treated in the 1970s (Table III). For men, the leukaemia risk more than doubled, from seven-fold to twenty-fold; the risk for women was about ten-fold, but with little change over time. An increase in risk between the 1960s and 1970s was seen for most sites of second cancer.

### *Ovarian cancer*

Of 13,044 women with cancer of the ovary, almost 10% (1242 women) were excluded from analysis (Table I); more than half of these (679, 5.2% of cohort) had had a previous tumour or a tumour registered in the same calendar year as the ovarian cancer. Of the 11,802 women analysed, fewer than half survived to the first anniversary of treatment (Table IIA), and less than a fifth (17%) survived five years. The women were followed for a total of 31,572 (mean 2.7) person-years at risk of a second tumour (Table IIB). The proportion of women treated with chemotherapy alone arose from 22% in the 1960s to 31% in the 1970s (Table IIC).

We observed 170 second malignancies among these women at least one year after their ovarian cancer, against 152.9 expected (Table IV), an 11% excess (RR=1.1, CI 1.0–1.3). Cancer of the breast accounted for more than half the overall excess of second cancer (47 *vs.* 38.1, RR=1.2, CI 1.0–1.6). Women treated for ovarian cancer in the 1970s had a higher risk of subsequent breast cancer (RR=1.4) than women treated in the 1960s (RR=1.1). The youngest women with ovarian cancer appeared to have a much greater risk of subsequent breast cancer (Table V): among women aged less than 40 at ovarian cancer, the risk was five-fold (3 *vs.* 0.58, RR=5.2, CI 1.7–12.3), but for women aged 40 and over, the observed excess was small (44 *vs.* 37.54, RR=1.2), and compatible with an unchanged risk. Although the numbers are small, there is a marked decline of breast cancer risk with increasing age at ovarian cancer ($\chi^2_1$ for trend 4.9, $P=0.01$). All the excess breast cancers arose in the first ten years of follow-up (41 *vs.* 29.22 within 10 years; 6 *vs.* 8.90 at ten or more years), and most of the significant downward trend in breast cancer risk with increasing age at ovarian cancer is contributed by these early second breast cancers.

There was a greater than three-fold excess of leukaemia following ovarian cancer (10 *vs.* 2.7; RR=3.7; CI 2.0–6.3). The relative risk of leukaemia increased markedly from 1.2 for women treated for ovarian cancer in the 1960s to 8.0 for those treated in the 1970s. Rectal cancer occurred more often than expected (12 *vs.* 7.2, RR=1.7, CI 1.0–2.7), as did cancers of the lung and colon. We observed only one contralateral ovarian cancer more than five years after the first, against 8.7 expected, the only second cancer in significant deficit. This deficit is more apparent than real, however, since many women will have had both ovaries removed at surgery for their ovarian cancer, but the registry

**Table III** Relative risk (RR) of second malignancy by calendar period of Hodgkin's disease

| | *Period of diagnosis of Hodgkin's disease* | | | | | | | | | |
|---|---|---|---|---|---|---|---|---|---|---|
| | *1961–69* | | | *1970–80* | | | *1961–80* | | | |
| No. of persons | | 1,331 | | | 1,639 | | | 2,970 | | |
| Person-years at risk[a] | | 6,423 | | | 5,948 | | | 12,371 | | |
| *Second malignancies*[b] | *Obs* | *Exp* | *RR* | *Obs* | *Exp* | *RR* | *Obs* | *Exp* | *RR* | *90% confidence interval* |
| Leukaemia | 4 | 0.47 | 8.5 | 6 | 0.37 | 16.2 | 10 | 0.84 | 11.9 | 6.5–20.2 |
| Lung | 7 | 5.28 | 1.3 | 13 | 4.10 | 3.2 | 20 | 9.38 | 2.1 | 1.4– 3.1 |
| Breast (F) | 1 | 2.33 | 0.4 | 1 | 2.06 | 0.5 | 2 | 4.09 | 0.5 | 0.1– 1.5 |
| Cervix (F) | 2 | 0.46 | 4.4 | 2 | 0.34 | 5.9 | 4 | 0.80 | 5.0 | 1.7–11.4 |
| All sites | 27 | 23.03 | 1.2 | 31 | 18.75 | 1.6 | 58 | 41.78 | 1.4 | 1.1– 1.7 |

[a]To 31 December 1981, excluding each person's first year of follow-up; [b]Obs=observed, Exp=expected.

**Table IV** Relative risk (RR) of second malignancy by calendar period of ovarian cancer

| | *Period of diagnosis of ovarian cancer* | | | | | | | | | |
|---|---|---|---|---|---|---|---|---|---|---|
| | *1961–69* | | | *1970–80* | | | *1961–80* | | | |
| No. of women | 5,321 | | | 6,481 | | | 11,802 | | | |
| Person-years at risk[a] | 15,202 | | | 8,811 | | | 24,013 | | | |
| *Second malignancies*[b] | *Obs* | *Exp* | *RR* | *Obs* | *Exp* | *RR* | *Obs* | *Exp* | *RR* | *90% confidence interval* |
| Leukaemia | 2 | 1.67 | 1.2 | 8 | 1.00 | 8.0 | 10 | 2.67 | 3.7 | 2.0–6.4 |
| Lung | 13 | 9.56 | 1.4 | 6 | 5.97 | 1.0 | 19 | 15.53 | 1.2 | 0.8–1.8 |
| Breast | 26 | 23.56 | 1.1 | 21 | 14.55 | 1.4 | 47 | 38.11 | 1.2 | 1.0–1.6 |
| Colon | 10 | 8.80 | 1.1 | 5 | 5.13 | 1.0 | 15 | 13.93 | 1.1 | 0.7–1.7 |
| Rectum | 5 | 4.53 | 1.1 | 7 | 2.66 | 2.6 | 12 | 7.19 | 1.7 | 1.0–2.7 |
| All sites | 96 | 90.06 | 1.1 | 74 | 62.88 | 1.2 | 170 | 152.94 | 1.1 | 1.0–1.3 |

[a]To 31 December 1981, excluding each person's first year of follow-up; [b]Obs = observed, Exp = expected.

**Table V** Second breast cancer, by age at ovarian cancer and time since ovarian cancer

| | *Time since diagnosis of ovarian cancer (years)* | | | | | | *All periods* | | | |
|---|---|---|---|---|---|---|---|---|---|---|
| | *1–4* | | *5–9* | | *10–21* | | *1–21* | | | |
| No. of women[a] | 5,341 | | 1,984 | | 1,057 | | 5,341 | | | |
| Person-years at risk[b] | 11,831 | | 7,231 | | 4,951 | | 24,013 | | | |
| | *Obs* | *Exp* | *Obs* | *Exp* | *Obs* | *Exp* | *Obs* | *Exp* | *RR* | *90% confidence interval* |
| Age at ovarian cancer (years) | | | | | | | | | | |
| <30 | 1 | 0.01 | – | 0.01 | – | 0.00 | 1 | 0.02 | 50.0 | 2.6–237.2 |
| 30–39 | 1 | 0.27 | 1 | 0.19 | – | 0.10 | 2 | 0.56 | 3.6 | 0.6– 11.2 |
| 40–49 | 6 | 2.53 | 1 | 1.25 | – | 0.70 | 7 | 4.48 | 1.6 | 0.7– 2.9 |
| 50–59 | 8 | 5.22 | 4 | 3.08 | 1 | 1.82 | 13 | 10.12 | 1.3 | 0.8– 2.0 |
| 60–69 | 9 | 5.36 | 3 | 3.65 | 3 | 2.99 | 15 | 12.00 | 1.2 | 0.8– 1.9 |
| 70–84 | 2 | 4.31 | 5 | 3.34 | 2 | 3.29 | 9 | 10.94 | 0.8 | 0.4– 1.4 |
| All ages (<85) | 27 | 17.70 | 14 | 11.52 | 6 | 8.90 | 47 | 38.12 | 1.2 | 1.0– 1.6 |
| RR | | 1.53 | | 1.22 | | 0.67 | | | | |

[a]No. of women still alive and at risk of breast cancer at the beginning of successive time intervals; [b]To 31 December 1981; excluding each woman's first year of follow-up.

records do not contain the data needed to obtain the appropriate risk (for women who still have at least one ovary). If we exclude ovarian cancer as a second tumour for this reason, the overall picture changes only slightly (169 *vs.* 144.2, RR = 1.2, CI 1.0–1.3).

*Testicular cancer*

Of 2,080 men registered with testicular cancer, 67 (3%) were excluded (Table I). Less than half of these (1,004, 48%) were still alive and at risk of a second cancer five years after treatment (Table IIA). The cohort was followed up for a total of 13,655 (mean 6.8) person-years at risk to the end of 1981 (Table IIB). Three per cent of men with testicular cancer were treated with chemotherapy alone in the 1960s, compared to 7% in the 1970s (Table IIC).

We observed 27 second cancers one or more years after testicular cancer, fewer than the 36.36 expected (RR = 0.7, CI 0.5–1.0) (Table VI). The only second tumours which occurred in significant excess were contralateral testicular tumours, of which there were five, each with a different histology from the first tumour. The overall risk was 8.1 (CI 3.6–16.0). The excess of leukaemia was small (2 *vs.* 0.80), and there was no excess of lung cancer. No significant deficit of second cancer was observed at any site. Apart from cancers of the contralateral testis, for which the risk rose from 2.9 to 14.3 over the two decades, there was no general increase in second cancer risk between testicular cancer patients treated in the 1960s and those treated in the 1970s (Table VI).

*Leukaemia as a second malignancy*

Leukaemia was consistently observed more often than expected after each of the three index tumours, in both decades, and in each sex (Table VII). Among the 18,262 subjects in all three cohorts, 22 second leukaemias were observed, against 4.41 expected, a five-fold overall risk (RR = 5.0, CI 3.4–7.1). Among the different types of leukaemia, the smallest excess risk was for lymphatic leukaemia (RR = 2.5). Leukaemia risk was greater than ten-fold for patients with Hodgkin's disease (RR = 11.9, CI 6.5–20.2), and more than three-fold for women with ovarian tumours (RR = 3.7, CI 2.0–6.3); the two-fold excess for men with testicular cancer was not significant. Fifteen (68%) of the 22 second leukaemias were acute, eleven (50%) of them acute myeloid leukaemia; only two (10%) were chronic lymphatic leukaemia.

**Discussion**

New primary cancers are perhaps the most serious late complication of cytotoxic chemotherapy for cancer or non-neoplastic disease (Calabresi, 1983; Anon, 1984; Whitehouse, 1985; Meadows & Hobbie, 1986; Kinlen *et al.*, 1979). Previous studies have considered mainly the risk of acute leukaemia after Hodgkin's disease (see Grunwald & Rosner, 1982) and ovarian cancer (Reimer *et al.*, 1977; Greene *et al.*, 1982; Haas *et al.*, 1987), but the risk of other second cancers has also been examined among patients with Hodgkin's disease (Brody & Schottenfeld, 1980; Boivin & Hutchison,

**Table VI** Relative risk (RR) of second malignancy by calendar period of testicular cancer

| | *Period of diagnosis of testicular cancer* | | | | | | | | | |
|---|---|---|---|---|---|---|---|---|---|---|
| | *1961–69* | | | *1970–80* | | | *1961–80* | | | |
| No. of men | 774 | | | 1,239 | | | 2,013 | | | |
| Person-years at risk[a] | 7,240 | | | 4,627 | | | 11,867 | | | |
| *Second malignancies*[b] | *Obs* | *Exp* | *RR* | *Obs* | *Exp* | *RR* | *Obs* | *Exp* | *RR* | *90% confidence interval* |
| Leukaemia | 2 | 0.51 | 3.9 | 0 | 0.29 | 0.0 | 2 | 0.80 | 2.5 | 0.4– 7.9 |
| Lung | 6 | 7.42 | 0.8 | 1 | 3.47 | 0.3 | 7 | 10.89 | 0.6 | 0.3– 1.2 |
| Testis | 1 | 0.34 | 2.9 | 4 | 0.28 | 14.3 | 5 | 0.62 | 8.1 | 3.2–17.0 |
| All sites | 19 | 24.10 | 0.8 | 8 | 12.10 | 0.7 | 27 | 36.20 | 0.7 | 0.5– 1.0 |

[a]To 31 December 1981, excluding each person's first year of follow-up; [b]Obs = observed, Exp = expected.

**Table VII** Relative risk (RR) of second leukaemia[a] by type of index primary tumour

| | | *Hodgkin's disease* | | *Ovary* | | *Testis* | | *All types* | | | |
|---|---|---|---|---|---|---|---|---|---|---|---|
| *ICD–8* | *Leukaemia type* | *Obs* | *Exp* | *Obs* | *Exp* | *Obs* | *Exp* | *Obs* | *Exp* | *RR* | *90% confidence interval* |
| 204 | Lymphoid | 1 | 0.34 | 3 | 0.96 | 0 | 0.29 | 4 | 1.59 | 2.5 | 0.9– 5.8 |
| 205 | Myeloid | 7 | 0.41 | 4 | 1.34 | 0 | 0.40 | 11 | 2.15 | 5.1 | 2.9– 8.5 |
| 206 | Monocytic | 2 | 0.04 | 0 | 0.13 | 0 | 0.03 | 2 | 0.20 | 10.0 | 1.8–31.5 |
| 207 | Other | 0 | 0.09 | 3 | 0.29 | 2 | 0.09 | 5 | 0.47 | 10.6 | 4.2–22.4 |
| 204–7 | All types | 10 | 0.88 | 10 | 2.72 | 2 | 0.81 | 22 | 4.41 | 5.0 | 3.4– 7.1 |
| | RR | 11.4 | | 3.7 | | 2.5 | | | | | |
| | (90% confidence interval) | (6.2–19.3) | | (2.0–6.2) | | (0.4–7.8) | | | | | |

[a]Obs = observed, Exp = expected.

1984; Henry-Amar, 1983) and testicular cancer (Hay *et al.*, 1984; Maatman *et al.*, 1984; Dieckmann *et al.*, 1986). Most of these studies were based on a series of several hundred or a few thousand patients recruited from one or several referral hospitals, although Henry-Amar's (1983) study included patients in a multi-centre trial. Risk estimates for leukaemia following Hodgkin's disease, not always adjusted for age, sex and time, ranged from 3- to 17-fold, but were often based on small numbers of cases. Leukaemia risk has been strongly associated with chemotherapy in these reports: 45 of 46 second leukaemias in the studies reviewed by Boivin and Hutchison (1984) arose in patients given chemotherapy with their first course of treatment, while the risk in patients treated without chemotherapy showed little increase over background rates.

This study at the South Thames Cancer Registry has shown that although the highest risk of second primary malignancy following treatment for Hodgkin's disease is for acute leukaemia, some solid tumours also occur in excess. Lung cancer risk was twice as high as expected after Hodgkin's disease, and there was a five-fold excess of cervix cancer. Both results are statistically significant, although the cervix result is based on only four observed cases. Smoking seems unlikely to be an important confounding factor in either case, since it is not known to be associated with Hodgkin's disease, and confounding is also an unlikely explanation for risks of this magnitude. Nor can the excess of cervical cancer be explained by social class, since the social class gradient for Hodgkin's disease, which is more common in higher socio-economic groups, is the reverse of that for cervical cancer.

The relative risk of any second primary malignancy after Hodgkin's disease increased from 1.2 to 1.6 between the 1960s and the 1970s, and the relative risk of leukaemia doubled from 8-fold to 16-fold. Increases in risk were seen for most individual sites of second cancer, although the numbers are mostly too small to reach statistical significance. This is not the result of improvement in survival in the later period, since the risk estimates take precise account of each subject's observed survival. The proportion of patients treated only with chemotherapy rose by half among men and by a quarter among women between the two decades covered by this study, and it is at least plausible that the increases in risk resulted from the use of more aggressive, multi-agent regimes which were introduced for Hodgkin's disease in the late 1960s and early 1970s.

The overall excess of second malignancy after cancer of the ovary is small, the largest contributions coming from breast cancer and leukaemia. The relative risk of leukaemia increased markedly between the decades, and there were smaller increases in risk for cancers of the rectum and breast. The proportion of women initially treated only with chemotherapy increased from 22% to 31% between the same periods. The mean duration of follow-up for women with ovarian cancer first seen in the 1970s was less than two years; the latency of most solid tumours is likely to be longer than this, and the possibility of a greater risk for solid tumours after longer periods of follow-up cannot be excluded for women treated in the 1970s.

The excess risk of breast cancer after cancer of the ovary was largely confined to women under the age of 40 at diagnosis of ovarian cancer, and the steep decrease in risk with age suggests that this may be due to higher susceptibility to radiation and chemical carcinogenesis in the breast tissue of younger women (Boice & Monson, 1977).

No significant excess of second cancer was seen among men with cancer of the testis, except for contralateral testicular cancer. The overall risk of a second cancer of the testis was 8.1, increasing sharply from three-fold for men treated in the 1960s to 14-fold for those treated in the 1970s.

Relatively few men with testicular cancer were treated with chemotherapy alone in either time period.

The excess risk of second leukaemia would undoubtedly have been even greater if all leukaemias diagnosed after a previous cancer had been registered. We have good evidence (Dr S. Nayfield, personal communication) that such leukaemias were often not registered at South Thames, because even if they were treated clinically as a new leukaemia, this diagnosis was not always explicitly recorded. This was particularly so if they supervened in patients with Hodgkin's disease, in which leukaemia or a leukaemic phase has often been considered to be part of the natural history of the disease (Reimer *et al.*, 1977). However, the striking increase in the risk of second leukaemia following Hodgkin's disease seen in the twenty years covered by this study suggests that the two diseases are probably quite distinct, and that the association between them requires a different explanation.

This study confirms that significant excess risks of second malignancy – both leukaemia and solid tumours – occur in patients with Hodgkin's disease and cancers of the ovary or testis, although the excess of solid tumours in testicular cancer patients was confined to contralateral tumours of the testis. Leukaemia risk was increased five-fold, and more than ten-fold among patients with Hodgkin's disease. There is a tendency towards higher excess risks of second malignancy in patients with Hodgkin's disease and ovarian cancer who were first treated in the 1970s, when cytotoxic agents were used as the mainstay of treatment more extensively than in the 1960s. No such general pattern was observed for men with testicular cancer; *cis*-platinum was introduced in the UK in 1978, and radiotherapy was the principal treatment recorded at registration in both time periods, but the risk of a second testicular cancer did increase between the decades. These patterns of risk suggest that at least part of the increase in second cancer risk observed between patients first treated for Hodgkin's disease and ovarian cancer in the 1960s and those treated in the 1970s may be related to chemotherapy. Case-control studies are now being carried out on lung cancer following Hodgkin's disease, and on leukaemia following each of the three index tumours considered here, and the detailed treatment records will enable any risks associated with chemotherapy to be carefully examined.

Despite widespread use of cytotoxic drugs in cancer therapy, the development of a second primary malignancy still remains an uncommon late complication, arising in perhaps 5 to 10% of patients. This may reflect the limited survival of many patients treated so far (Whitehouse, 1985), and as survival begins to approach or exceed the induction period of solid tumours, these risks seem likely to increase (Kaldor *et al.*, 1987). It may be possible to reduce the carcinogenicity of cancer therapy without loss of efficacy, either by reduction in dose or duration, or perhaps by modification of anti-cancer drugs themselves.

There is a need for continuous monitoring of the late effects of cancer therapy, along the lines of the Late Effects Study Group for childhood tumours in the USA (Tucker *et al.*, 1984). Cancer chemotherapy regimes are frequently revised: the pace of these changes and the complexity of the regimes themselves will make it difficult to assess any cancer risks associated with a particular drug or regime unless a deliberate and systematic effort is made to record second cancers, and to identify them clearly as such in the hospital records, from which cancer registrations are ultimately derived. The number of second primary malignancies recorded by a single regional cancer registry will usually be insufficient to enable the risks associated with different cancer treatment regimes to be clearly distinguished, but a collaborative group of cancer registries in the UK with an interest in the late effects of cancer treatment, using both cohort and case-control methods, would be able to provide early, precise and unbiased estimates of risk from particular cytotoxic regimes. This would contribute toward improved safety of cancer therapy and to reduction of cancer risk in the population.

We are grateful for advice and assistance from Mr Richard Skeet, Director of the Thames Cancer Registry.

### References

ANON. (1984). Drugs that can cause cancer. *Lancet*, **i**, 261.

ANON. (1985). Second malignancies in lymphoma patients. *Lancet*, **ii**, 1163.

BERRY, G. (1983). The analysis of mortality by the subject-years method. *Biometrics*, **39**, 173.

BOICE, J.D. & MONSON, R.R. (1977). Breast cancer in women after repeated fluoroscopic examinations of the chest. *J. Natl Cancer Inst.*, **59**, 823.

BOICE, J.D., GREENE, M.H., KILLEN, J.Y. & 5 others (1983). Leukaemia and pre-leukaemia after adjuvant treatment of gastro-intestinal cancer with semustine (methyl CCNU). *N. Engl. J. Med.*, **309**, 1079.

BOIVIN, J.F. & HUTCHISON, G.B. (1984). Second cancers after treatment for Hodgkin's disease: A review. In *Radiation carcinogenesis: Epidemiology and biological significance*, Boice, J.D. & Fraumeni, J.F., Jr., (eds) p. 181. Raven Press: New York.

BRESLOW, N.E. (1984). Elementary methods of cohort analysis. *Int. J. Epidemiol.*, **13**, 112.

BRODY, R.S. & SCHOTTENFELD, D. (1980). Multiple primary cancers in Hodgkin's disease. *Sem. Oncol.*, **7**, 187.

CALABRESI, P. (1983). Leukemia after cytotoxic chemotherapy – a pyrrhic victory? *N. Engl. J. Med.*, **309**, 1118.

COLEMAN, M., DOUGLAS, A., HERMON, C. & PETO, J. (1986). Cohort study analysis with a FORTRAN computer program. *Int. J. Epidemiol.*, **15**, 134.

CURTIS, R.E., HANKEY, B.F., MYERS, M.H. & YOUNG, J.L. (1984). Risk of leukaemia associated with the first course of cancer treatment: An analysis of the surveillance, epidemiology and end results program experience. *J. Natl Cancer Inst.*, **72**, 531.

DIECKMANN, K-P., BOECKMANN, W., BROSIG, W., JONAS, D. & BAUER, H.W. (1986). Bilateral testicular germ cell tumours: Report of 9 cases and review of the literature. *Cancer*, **57**, 1254.

GREENE, M.H., BOICE, J.D., GREER, B.E., BLESSING, J.A. & DEMBO, A.J. (1982). Acute nonlymphocytic leukaemia after therapy with alkylating agents for ovarian cancer. A study of five randomised controlled clinical trials. *N. Engl. J. Med.*, **307**, 1416.

GRUNWALD, H.W. & ROSNER, F. (1979). Acute leukaemia and immunosuppressive drug use: A review of patients undergoing immunosuppressive therapy for non-neoplastic disease. *Arch. Intern. Med.*, **139**, 461.

GRUNWALD, H.W. & ROSNER, F. (1982). Acute myeloid leukaemia following treatment of Hodgkin's disease. *Cancer*, **50**, 676.

HAAS, J.F., KITTELMANN, B., MEHNERT, W. & 4 others (1987). Risk of leukaemia in ovarian tumour and breast cancer patients following treatment by cyclophosphamide. *Br. J. Cancer*, **55**, 213.

HAY, J.H., DUNCAN, W. & KERR, G.R. (1984). Subsequent malignancies in patients irradiated for testicular tumours. *Br. J. Radiol.*, **57**, 597.

HENRY-AMAR, M. (1983). Second cancers after radiotherapy and chemotherapy for early stages of Hodgkin's disease. *J. Natl Cancer Inst.*, **71**, 911.

INTERNATIONAL AGENCY FOR RESEARCH ON CANCER (1982). *Carcinogenic risk of chemicals in humans*. Monograph supplement 4, IARC: Lyon.

KALDOR, J.M., DAY, N.E., BAND, P. & 11 others (1987). Second malignancies following testicular cancer, ovarian cancer and Hodgkin's disease: An international collaborative study among cancer registries. *Int. J. Cancer*, **39**, 571.

KENNEDY, B.J., LOEB, V., PETERSON, V.M., DONEGAN, W.L., NATARAJAN, N. & MELLTIN, C. (1985). National survey of patterns of care for Hodgkin's disease. *Cancer*, **56**, 2547.

KINLEN, L.J., SHEIL, A.G.R., PETO, J. & DOLL, R. (1979). Collaborative UK–Australian study of cancer in patients treated with immunosuppressive drugs. *Br. Med. J.*, **ii**, 1461.

KRAUSE, J.R., AYUYANG, H.Q. & ELLIS, L.D. (1985). Secondary non-hematopoietic cancers arising following treatment of hematopoietic disorders. *Cancer*, **55**, 512.

MAATMAN, T., BUKOWSKI, R.M. & MONTIE, J.E. (1984). Retroperitoneal malignancies several years after initial treatment of germ cell cancer of the testis. *Cancer*, **54**, 1962.

McVIE, J.G. & SOMERS, R. (1985). Chemotherapy of Hodgkin's disease comes of age. *Br. Med. J.*, **290**, 950.

MEADOWS, A.T. & HOBBIE, W.L. (1986). The medical consequences of cure. *Cancer*, **58**, 524.

NATIONAL INSTITUTES OF HEALTH (1986). National Institutes of Health Consensus Development Conference Statement: Adjuvant chemotherapy for breast cancer. September 1985 *CA-A Cancer Journal for clinicians*, **36**, 42. (*Conference Report*).

NEWLANDS, E.S., BEGENT, R.H.J., RUSTIN, G.J.S., PARKER, D. & BAGSHAWE, K.D. (1983). Further advances in the management of malignant teratomas of the testis and other sites. *Lancet*, **i**, 948.

PAYNE, G. (1976). UK, England, South Metropolitan Region. In *Cancer Incidence in Five Continents*. **Vol. III.** Waterhouse, J.A.H. *et al* (eds) p. 388 (IARC Sci. Publ. no. 15). IARC: Lyon.

REIMER, R.R., HOOVER, R., FRAUMENI, J.F., Jr. & YOUNG, R.C. (1977). Acute leukaemia after alkylating-agent therapy of ovarian cancer. *N. Engl. J. Med.*, **297**, 177.

ROTHMAN, K.J. & BOICE, J.D. (1982). *Epidemiologic analysis with a programmable calculator*. Epidemiology Resources Inc: Boston.

SCHMAHL, D. (1986). Carcinogenicity of anticancer drugs and especially alkylating agents. In *Carcinogenicity of Alkylating Cytostatic Drugs*, Schmahl D. & Kaldor, J.M. (eds) (IARC Sci. Publ. no. 78). IARC: Lyon.

SKEET, R.G. (1982). UK, England, South Thames Region. In *Cancer Incidence in Five Continents*. **Vol. IV**. Waterhouse, J.A.H. *et al.* (eds) p. 562. (IARC Sci. Publ. no. 42). IARC: Lyon.

TUCKER, M.A., MEADOWS, A.T., BOICE, J.D., HOOVER, R.N. & FRAUMENI, J.F. (1984). Cancer risk following treatment of childhood cancer. In *Radiation Carcinogenesis: Epidemiology and Biological Significance*, Boice, J.D., Jr. & Fraumeni, J.F., Jr. (eds) p. 211. Raven Press: New York.

WHITEHOUSE, J.M.A. (1985). Risk of leukaemia associated with cancer chemotherapy. *Br. Med. J.*, **290**, 261.

WORLD HEALTH ORGANIZATION (1967). *International Statistical Classification of Injuries, Diseases and Causes of Death*. 8th revision, **Vol. I**. WHO: Geneva.